# Supplementary figures and images for: Stratifying ALS Patients by Mode of Inheritance Reveals Transcriptomic Signatures Specific to sALS and fALS
Source: Int J Mol Sci. 2025 Sep 22;26(18):9234. doi: 10.3390/ijms26189234 (PMC12470382; doi:10.3390/ijms26189234)

Motor Cortex Module–Trait Relationships  
Heatmap scale: signed bicor r-value

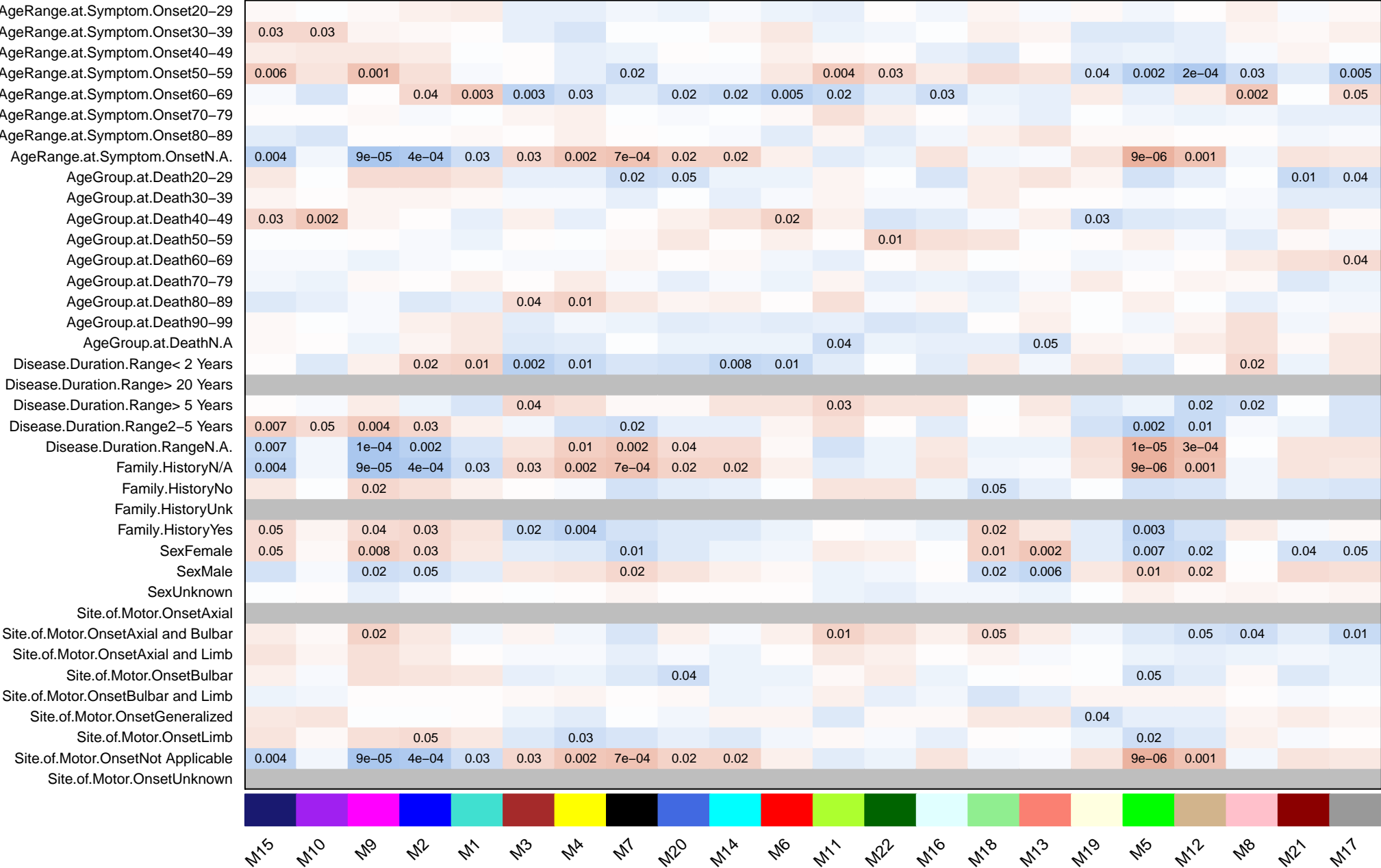

Supplement: Supplementary file 1 [file ijms-26-09234-s001.zip › SupplTable S1-Correlation Heatmap Motor Cortex.pdf]

**Lumbar Spinal Cord Module–Trait Relationships**  
Heatmap scale: signed bicor r-value

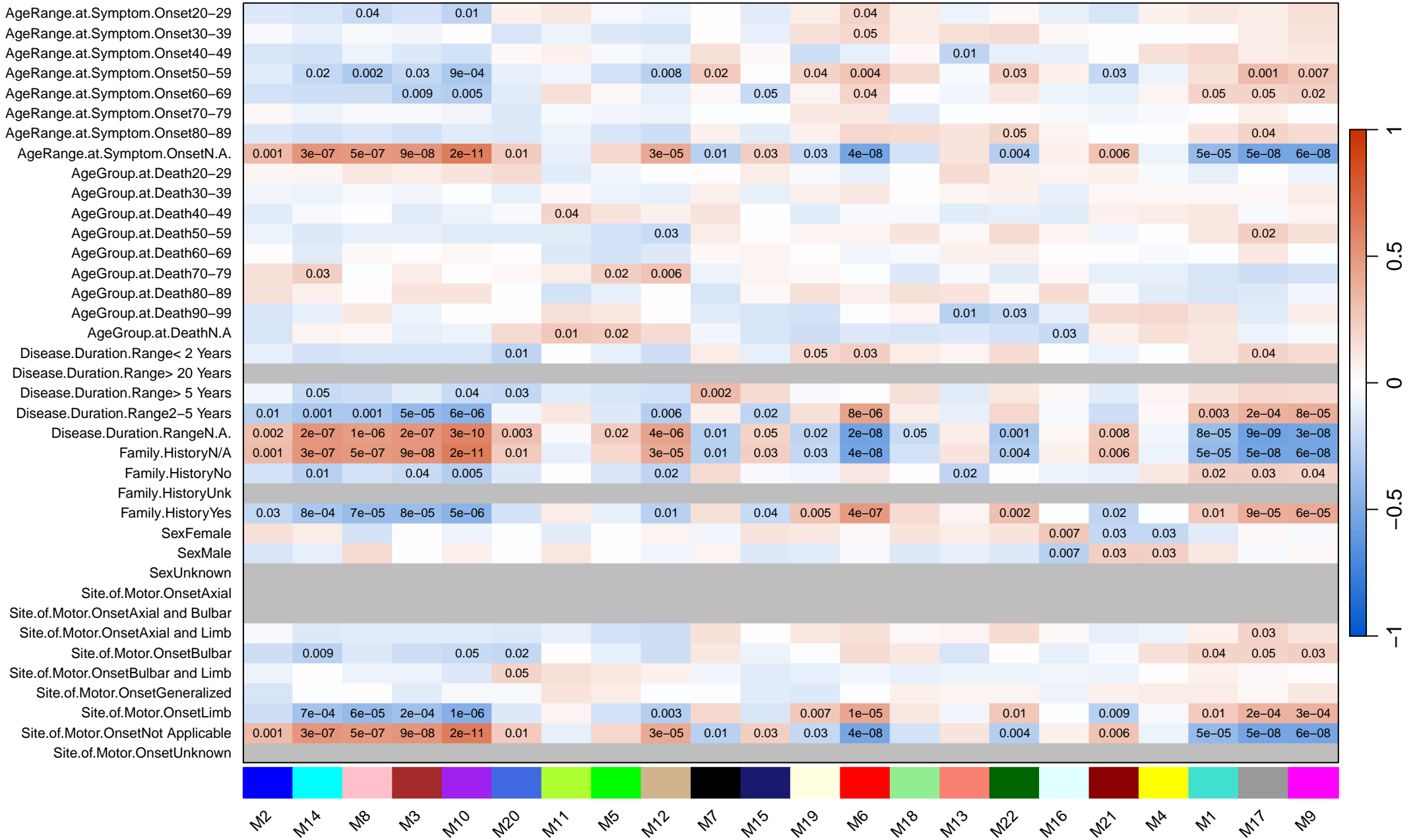

Supplement: Supplementary file 1 [file ijms-26-09234-s001.zip › SuppTable S2-Correlation Heatmap Lumbar Spinal Cord.pdf]
